# The effectiveness of tobacco control television advertisements in increasing the prevalence of smoke-free homes

**DOI:** 10.1186/s12889-015-2207-2

**Published:** 2015-09-08

**Authors:** S. Lewis, M. Sims, S. Richardson, T. Langley, L. Szatkowski, A. McNeill, A. B. Gilmore

**Affiliations:** UK Centre for Tobacco and Alcohol Studies, Division of Epidemiology and Public Health, University of Nottingham, Clinical Sciences Building, Nottingham City Hospital, Nottingham, NG5 1PB UK; UK Centre for Tobacco and Alcohol Studies, Department for Health, University of Bath, Bath, BA2 7AY UK; UK Centre for Tobacco and Alcohol Studies, Institute of Psychiatry, King’s College London, 16 de Crespigny Park, London, SE5 8AF UK

## Abstract

**Background:**

There is considerable evidence that tobacco control mass media campaigns can change smoking behaviour. In the UK, campaigns over the last decade have contributed to declines in smoking prevalence and been associated with falls in cigarette consumption among continuing smokers. However, it is less evident whether such campaigns can also play a role in changing smokers’ behaviour in relation to protecting others from the harmful effects of their smoking in the home. We investigated whether exposure to English televised tobacco control campaigns, and specifically campaigns targeting second hand smoking, is associated with smokers having a smoke-free home.

**Methods:**

We used repeated cross-sectional national survey data on 9872 households which participated in the Health Survey for England between 2004 and 2010, with at least one adult current smoker living in the household. Exposure to all government-funded televised tobacco control campaigns, and to those specifically with a second hand smoking theme, was quantified in Gross Rating Points (GRPs), an average per capita measure of advert exposure where 100 GRPs indicates 100 % of adults exposed once or 50 % twice. Our outcome was self-reported presence of a smoke-free home (where no one smokes in the home on most days). Analysis used generalised additive models, controlling for individual factors and temporal trends.

**Results:**

There was no association between monthly televised campaigns overall and the probability of having a smoke-free home. However, exposure to campaigns specifically targeting second hand smoke was associated with increased odds of a smoke-free home in the following month (odds ratio per additional 100 GRPs, 1.07, 95 % CI 1.01 to 1.13), though this association was not seen at other lags. These effects were not modified by socio-economic status or by presence of a child in the home.

**Conclusions:**

Our findings provide tentative evidence that mass media campaigns specifically focussing on second hand smoke may be effective in reducing smoking in the home, and further evaluation of campaigns of this type is needed. General tobacco control campaigns in England, which largely focus on promoting smoking cessation, do not impact on smoke-free homes over and above their direct effect at reducing smoking.

## Background

Secondhand smoke (SHS) exposure is a serious danger to health [1], and children are particularly vulnerable [2]. Globally, approximately 600,000 deaths a year, 28 % of them in children, result from non-smokers’ involuntary exposure to other people’s tobacco smoking [3]. The numerous diseases caused by SHS mimic those caused by active smoking and include, in adults, cardiovascular disease and lung cancer, and in children, sudden infant death as well as a range of respiratory and other illnesses [1]. In jurisdictions which have introduced smoke-free legislation which prohibits smoking in enclosed public places, the predominant place of exposure for children and most non-smoking adults is now the home [1]. The priority now for public health practitioners and policy makers is therefore to reduce exposure, especially of children, in the home. People who live in “smoke-free homes” - that is, homes where smokers only smoke outside the home - have much lower levels of SHS exposure [4]. Smokers living in smoke-free homes are also more likely to attempt to quit [5, 6], to succeed in doing so [5–8], are less likely to relapse [6–8], and their children may be less likely to take up smoking themselves [9]. Yet, whilst it is known that smokers are more likely to make their home smoke-free if they have young children, live with a non-smoking adult [5, 6, 10], or are relatively socially advantaged [11], there are to date few insights into how to encourage more smokers to make their homes smoke-free. A range of household and individual-level interventions have been proposed and tested but few have been effective, and these tend to have been intensive interventions which may not be cost effective [12]. From a theoretical standpoint, it has been argued that what is needed is to make control of SHS around children more socially acceptable and eventually the norm, and that this may be more effectively achieved through population-based strategies [13].

At the population level, there is some evidence that having a comprehensive tobacco control programme is associated with a higher prevalence of smoke-free homes [14]; for example, prevalence of smoke-free homes is seen to vary across US and Australian states in line with the comprehensiveness of tobacco control programmes implemented. Mass media campaigns are an important component of most tobacco control programmes. Research with smokers in the UK shows that whilst the majority are aware that SHS can be harmful, they underestimate the real risks to children’s and adults’ health [10]. There is some limited evidence that mass media campaigns can change knowledge and attitudes about SHS [10, 15, 16]. Furthermore, there is also now considerable evidence that mass media campaigns can change smoking behaviour [17, 18]; in the UK we have recently shown that campaigns over the last decade have contributed to declines in smoking prevalence and have been associated with falls in cigarette consumption in those who continue to smoke [19]. However, it is less evident whether such campaigns can also play a role in changing smokers’ behaviour in relation to protecting others from the harmful effects of their smoking in the home.

This paper therefore used repeated cross-sectional data from a large national survey to investigate whether televised government-funded tobacco control campaigns - both overall and those specifically aimed at influencing smokers’ knowledge and behaviour in relation to the effects of their smoking on others - resulted in an increase in the number of smokers maintaining a smoke-free home in England.

## Methods

### Survey data

We used data from the Health Survey for England, from January 2004 to April 2010 inclusive. This is an annual cross-sectional survey designed to be representative of adults and children living in private households in England [20]. A sample of adults and children is drawn each year using a clustered, stratified, multistage design. This involves selecting a random sample of postcode sectors (the primary sampling units; PSUs) with probability proportional to the total number of addresses within them. PSUs are stratified before selection by two variables: local authority (government boundaries) and proportion of households in the 2001 Census with a head of household with a non-manual occupation (NS-SEC groups 1–3). Within each selected PSU, a random sample of postal addresses is then selected. Once selected, PSUs are randomly allocated to the 12 months of the year for the interview to be conducted. The Health Survey for England data is sponsored by the Information Centre for Health and Social Care and the Department of Health, and made freely available in an anonymised form to registered users through the UK Data Archive [http://www.data-archive.ac.uk/].

At each co-operating eligible household, the interviewer first completed a household questionnaire, with information obtained from the household reference person or their partner. An individual interview was then carried out with all adults aged 16 years old and over and with up to two children in each household.

The trend in number of smoke-free homes is in part determined by smoking rates (a household of non-smokers is significantly more likely to have a smoke-free home [21]). To avoid the indirect effect television advertisements may have on the prevalence of smoke-free homes via its influence on smoking rates that we have previously demonstrated [19], we restricted our analysis to households with at least one adult smoker (aged 18 and over). Information on month and year of interview was used to match the survey data to campaign exposure data.

Adults were defined as smokers if they responded ‘Yes’ when asked “*Do you smoke cigarettes at all nowadays?”* A home was defined as smoke-free if the respondent completing the household questionnaire said ‘No’ to the question: “*Does anyone smoke inside the home on most days*”.

### Campaign exposure

Exposure to government-funded national televised tobacco control campaigns, or those run by charities such as the British Heart Foundation and Cancer Research UK but funded by the Department of Health, was quantified in Gross Rating Points (GRPs). GRPs are a standard broadcasting industry measure of advertising exposure, commonly used in evaluations of televised mass media campaigns. Television viewer figures at the time when the advertisements are shown are collected by the Broadcasters' Audience Research Board via a metered panel, and GRPs combine reach and frequency and are equivalent to the summed ratings of individual advertisements [television ratings (TVRs)]. GRPs are a population-averaged indicator of exposure, for example, 100 GRPs could indicate that 100 % of adults were exposed to an advertisement once, or that 50 % were exposed twice. They do not provide a measure actual exposure on the individual-level, which would be dependent on an individual’s time, channel and frequency of television viewing. We categorised campaign types according to their theme, content and style using their video recordings and/or creatives, described in detail elsewhere [22]. As part of this coding process, campaigns were categorised as focusing on a second hand smoking theme, or other theme. Campaigns with a second hand smoking theme included the ‘Second hand smoke is a killer’ campaign which aimed to show smokers the health effects that SHS can have on adults that are around the smoker and the ‘Invisible killer’ campaign which aimed to show the hidden dangers of SHS on both young and old, in particular that 85 % is invisible and odourless. Other campaigns predominantly had a smoking cessation theme. For each month, we then summed GRPs for each of these two campaign themes to derive time series of monthly GRPs for each.

### Statistical analysis

We analysed the association between overall exposure to televised tobacco control campaigns, and exposure to the two types of campaign themes, on the probability of a household with at least one adult smoker being smoke-free. We used binary logistic generalised additive (GAM) models in the statistical package R using the gamm4 function [23]. These models allow us to fit non-linear effects of exposures. The effects of GRP exposures were initially considered as non-linear effects, specifically cubic restricted splines, and the effective degrees of freedom (edf) was used to assess linearity. All these effects were found to be linear (i.e., the edf obtained was not significantly different to 1) and were subsequently fitted as linear terms, expressing exposure in units of increasing 100 GRPs per month. Since evidence suggests that tobacco control campaigns have their effects on smoking behaviour while campaigns are being broadcast and for a short time afterwards, we assessed the effects on current smoke-free home status of exposure in the same month, and exposure in the two previous months using lag terms in each model.

To allow for the sampling design, we adjusted for the stratification factors, Government office region and the NS-SEC (National Statistics Socio-economic Classification) of the household reference person in the model, and fitted the cluster indicator (PSU) as a random effect. Furthermore it was possible that the unequal selection probabilities for sampling postcode sectors might be correlated with the outcome variable and therefore induce bias in estimators of model parameters in this multi-level model [24]. We therefore included a further variable in our multi-level models representing the number of addresses in each postcode sector provided by NatCen Social Research [http://www.natcen.ac.uk/our-research/research/health-survey-for-england/] (who deliver the Health Survey for England) to control for this.

We also adjusted for a number of other household-level determinants of smoke-free homes, which were considered as possible confounders. These included measures of the number of smokers in the household, gender composition of smokers in the household, average age of smokers in the household, the average level of dependence of smokers in the household (determined using the Heaviness of Smoking Index for individual smokers averaged across all smokers in the household) [25], age of the youngest child in the household, household Index of Multiple Deprivation (IMD) score [http://webarchive.nationalarchives.gov.uk/20100410180038/http://communities.gov.uk/communities/neighbourhoodrenewal/deprivation/deprivation07/], and season of questionnaire, all coded as categorical variables.

We also adjusted for a monthly time trend. Although this was initially fitted as a non-linear effect using a thin plate regression spline term, the trend was found to be linear, and was subsequently fitted as a linear term in all models. We additionally adjusted for a measure of the extent of other current tobacco policies in England from 2004 to 2010 based on the Tobacco Control Scale (TCS) developed by Joossens and Raw [26], including a step increase in relation to the introduction of smoke-free legislation, but omitting scores relating to price, and operationalised as a four-level categorical variable for increasing tobacco control activity over time. In a sensitivity analysis, we also adjusted for population level smoking prevalence, as estimated from the Health Survey for England data, as an alternative marker of the effects of population-level smoking cessation interventions. We fitted interaction terms into our final models between socio-economic indicators (NS-SEC classification and IMD score) and campaign GRPs to determine whether socio-economic status might modify the effect of campaign exposures, and also fitted interaction terms with the presence of a child in the home to assess whether this may modify the effect of campaign exposures.

## Results

**Fig. 1:**
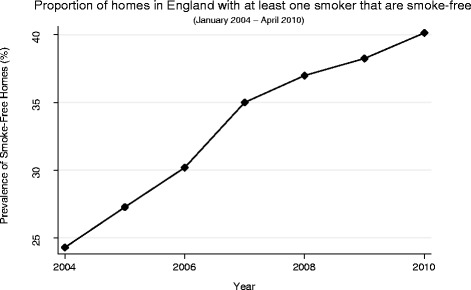
Proportion of households with at least one smoker that are smoke-free (January 2004 to April 2010)

**Fig. 2:**
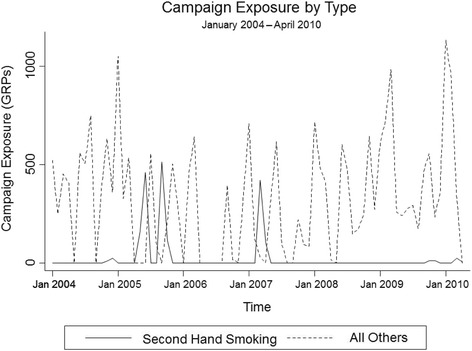
Time series of monthly campaign exposures by campaign type

Between 2004 and 2010, the response rate for the Health Survey for England varied between 64 % and 74 %. Of the 9,872 households interviewed with at least one smoker aged 18 or over 3,181 (32.2 %) reported being smoke-free (Fig. 1). The prevalence of smoke-free homes in our sample was found to increase over time (Fig. 1). Over this timeframe, the mean monthly exposure for all campaigns was 344.7 GRPs, ranging from a minimum of 0 to a maximum of 1,135.2 GRPs per month. GRPs specifically on the second hand smoking theme were low, occurring in only 12 of the 75 months in our study period, with a mean of 155.2 GRPs in the months that they occurred, ranging from a minimum of 0 to a maximum of 514.6 GRPs per month (Fig. 2).

The characteristics of the sample are shown in Table 1. In our multivariable models of the effects of tobacco control campaigns, overall (Table 2), and those specific to second hand smoking themes (Table 3), households that were more socioeconomically deprived (as measured by IMD or by socioeconomic status of the head of the household) were less likely to report being smoke-free. Households with children, where smokers were younger, where all smokers were male, and where smokers had lower levels of nicotine addiction, were more likely to report being smoke-free. The odds of a household being smoke-free increased over time in a linear fashion, and homes were more likely to be smoke-free in the summer than in the winter. The odds of a smoke-free home tended to increase with tobacco control score, though not significantly so in our final model for all campaigns (Table 2).

**Table 1:** Sample characteristics (2004–2010)

Covariate	Categories	Number	Percent
Total sample		9,872	100
Government office region	North East	719	7.3
	North West	1,508	15.3
	Yorkshire and the Humber	1,092	11.1
	East Midland	1,007	10.2
	West Midland	1,038	10.5
	East of England	1,041	10.5
	London	1,126	11.4
	South East	1,449	14.7
	South West	892	9.0
Gender of smokers in household	All female smokers	4,450	45.1
	All male smokers	3,679	37.3
	Mixed smokers	1,743	17.7
Average age of smokers in household	18–24	833	8.4
	25–39	3,396	34.4
	40–54	2,970	30.1
	55+	2,673	27.1
NS-SEC of head of household	Managerial & professional	2,675	27.1
	Intermediate	1,917	19.4
	Routine & manual	4,914	49.8
	Other	354	3.6
Average level of dependence of smokers in household^a^	0 (least addicted)	2,661	27.0
	1	1,409	14.3
	2	2,085	21.1
	3	1,958	19.8
	4	1,316	13.3
	5	324	3.3
	6 (most addicted)	119	1.2
Age of youngest child in household	No child	1,786	18.1
	0–5	1,401	14.2
	6–15	6,685	67.7
Number of adult smokers	Two or more smokers	3,475	35.2
	Lone smoker	4,372	44.3
	Lone smoker (lives alone)	2,025	20.5
Index of Multiple Deprivation	1 (least deprived)	1,346	13.6
	2	1,602	16.3
	3	1,870	18.9
	4	2,363	23.9
	5 (most deprived)	2,691	27.3
Season	Summer (Jun–Aug)	2,721	27.6
	Autumn (Sep–Nov)	2,560	25.9
	Spring (Mar–May)	2,674	27.1
	Winter (Dec–Feb)	2,367	24.0

Figures show baseline for categorical variables. ^a^The HSE surveys include two measures of nicotine dependence: cigarette consumption and time to first cigarette. Dependence score for a smoker was derived using these measures and scored based on the Modified Fagerstrom Test for Nicotine Dependence. A household measure of dependence was derived by averaging the score across all smokers in the household

**Table 2:** Effect of all tobacco control campaigns (2004–2010) and other factors on odds of smoke-free home, *n* = 9,872

Covariate	Categories	OR (95 % CI)	*p*
Time^a^		1.01 (1.01–1.02)	<0.001
Tobacco control campaigns	Total GRPs^a^	1.01 (0.99–1.04)	0.184
	Total GRPs (1 month)^a^	1.00 (0.98–1.02)	0.959
	Total GRPs (2 months)^a^	1.00 (0.98–1.02)	0.921
TCS Score	24.5	1	
	27	1.07 (0.84–1.36)	0.595
	48	1.41 (0.95–2.10)	0.092
	51	1.14 (0.74–1.74)	0.548
Season	Summer	1	
	Autumn	0.98 (0.84–1.13)	0.756
	Spring	0.88 (0.74–1.04)	0.135
	Winter	0.84 (0.71–0.98)	0.030
Government office region	North East	1	
	North West	0.67 (0.52–0.87)	0.027
	Yorkshire and the Humber	0.78 (0.59–1.02)	0.0366
	East Midland	0.71 (0.54–0.93)	0.013
	West Midland	0.85 (0.65–1.12)	0.246
	East of England	0.92 (0.70–1.20)	0.526
	London	0.71 (0.54–0.92)	0.010
	South East	0.73 (0.56–0.95)	0.017
	South West	1.09 (0.82–1.43)	0.557
Gender of smokers in household	All female smokers	1	
	All male smokers	1.43 (1.29–1.60)	<0.001
	Mixed smokers	1.18 (0.87–1.61)	0.276
Average age of smokers in household	18–24	1	
	25–39	0.74 (0.62–0.87)	<0.001
	40–54	0.52 (0.43–0.62)	<0.001
	55+	0.32 (0.26–0.39)	<0.001
NS-SEC of head of household	Managerial & professional	1	
	Intermediate	0.79 (0.69–0.91)	<0.001
	Routine & manual	0.64 (0.57–0.72)	<0.001
	Other	0.58 (0.43–0.78)	<0.001
Average level of dependence of smokers in household	0 (least addicted)	12.59 (6.72–23.61)	<0.001
	1	5.03 (2.67–9.48)	<0.001
	2	3.46 (1.84–6.51)	<0.001
	3	2.26 (1.20–4.25)	<0.012
	4	1.25 (0.66–2.37)	0.501
	5	0.42 (0.19–0.94)	0.034
	6 (most addicted)	1	
Age of youngest child in household	No child	1	
	0-5	2.59 (2.24–3.00)	<0.001
	6-15	1.34 (1.18–1.53)	<0.001
Number of adult smokers	Two or more smokers	1	
	Lone smoker	2.83 (2.12–3.78)	<0.001
	Lone smoker (lives alone)	0.84 (0.63–1.11)	0.223
Index of Multiple deprivation	1 (least deprived)	1	
	2	1.02 (0.86–1.21)	0.847
	3	0.79 (0.66–0.93)	0.006
	4	0.60 (0.50–0.71)	<0.001
	5 (most deprived)	0.41 (0.34–0.49)	<0.001

^a^Time and GRPs at different lags were initially considered as nonlinear smooth terms and as they were found to be linear (spline effective degrees of freedom = 1), replaced with linear terms. The table presents the ORs for having a smoke-free home associated with a 100 point increase in GRPs. Also included in the model is a covariate for number of addresses in each PSU. Likelihood ratio test *p* values are not shown for categorical variables as modelling was based on quasi-likelihood

**Table 3:** Effect of second hand smoking campaigns (2004–2010) on odds of smoke-free home, *n* = 9,872

Covariate	Categories	OR (95 % CI)	*p*
Time^a^		1.01 (1.01–1.02)	0.005
Tobacco control campaigns	Second hand smoke GRPs^a^	0.99 (0.93–1.05)	0.740
	Second hand smoke GRPs (1 month)^a^	1.07 (1.01–1.13)	0.033
	Second hand smoke GRPs (2 months)^a^	0.98 (0.92–1.04)	0.490
	Other GRPs^a^	1.02 (0.99–1.04)	0.134
	Other GRPs (1 month)^a^	1.00 (0.98–1.02)	0.755
	Other GRPs (2 months)^a^	1.01 (0.98–1.03)	0.561
TCS Score	24.5	1	
	27	1.13 (0.88–1.46)	0.334
	48	1.53 (1.02–2.31)	0.041
	51	1.25 (0.81–1.94)	0.320
Season	Summer	1	
	Autumn	0.97 (0.84–1.13)	0.695
	Spring	0.86 (0.73–1.02)	0.098
	Winter	0.84 (0.71–0.99)	0.041
Government office region	North East	1	
	North West	0.67 (0.52–0.87)	0.003
	Yorkshire and the Humber	0.78 (0.59–1.02)	0.069
	East Midland	0.71 (0.54–0.93)	0.014
	West Midland	0.85 (0.65–1.12)	0.246
	East of England	0.91 (0.70–1.20)	0.514
	London	0.71 (0.54–0.92)	0.010
	South East	0.73 (0.56–0.94)	0.017
	South West	1.08 (0.82–1.43)	0.567
Gender of smokers in household	All female smokers	1	
	All male smokers	1.44 (1.29–1.60)	<0.001
	Mixed smokers	1.18 (0.87–1.60)	0.280
Average age of smokers in household	18–24	1	
	25–39	0.73 (0.62–0.87)	<0.001
	40–54	0.51 (0.43–0.62)	<0.001
	55+	0.32 (0.26–0.38)	<0.001
NS-SEC of head of household	Managerial & professional	1	
	Intermediate	0.79 (0.69–0.91)	0.001
	Routine & manual	0.64 (0.57–0.72)	<0.001
	Other	0.58 (0.43–0.77)	<0.001
Average level of dependence of smokers in household	0 (least addicted)	12.51 (6.67–23.45)	<0.001
	1	5.00 (2.65–9.42)	<0.001
	2	3.43 (1.83–6.46)	<0.001
	3	2.25 (1.19–4.23)	0.012
	4	1.24 (0.65–2.35)	0.512
	5	0.42 (0.19–0.93)	0.033
	6 (most addicted)	1	
Age of youngest child in household	No child	1	
	0-5	2.60 (2.24–3.01)	<0.001
	6-15	1.34 (1.17–1.53)	<0.001
Number of adult smokers	Two or more smokers	1	
	Lone smoker	2.82 (2.12–3.77)	<0.001
	Lone smoker (lives alone)	0.83 (0.63–1.11)	0.217
Index of Multiple deprivation	1 (least deprived)	1	
	2	1.02 (0.86–1.21)	0.839
	3	0.79 (0.66–0.93)	0.006
	4	0.60 (0.50–0.71)	<0.001
	5 (most deprived)	0.41 (0.34–0.50)	<0.001

^a^Time and GRPs at different lags were initially considered as nonlinear smooth terms and as they were found to be linear (spline effective degrees of freedom = 1), replaced with linear terms. The table presents the ORs for having a smoke-free home associated with a 100 point increase in GRPs. Also included in the model is a covariate for number of addresses in each PSU. Likelihood ratio test *p* values are not shown for categorical variables as modelling was based on quasi-likelihood

We found no association between overall GRPs from all campaigns and odds that a given home was smoke-free. During the period 2004–2010, for every additional 100 GRPs of exposure to all televised tobacco control campaigns in the same month, there was a non-significant 1 % increase in the odds that a given household was smoke-free (OR: 1.01, 95 % CI: 0.99–1.04), as shown in Table 2. Neither the one or two-month lag terms were found to be statistically significant.

When campaign exposure was classified as that specific to a second hand smoking theme or otherwise, there was a significant positive association between exposure to campaigns with a second hand smoking theme at a one-month lag and the odds that a given household was smoke-free. For each additional 100 GRPs in exposure to these campaigns, we found a 7 % in the odds that a given household was smoke-free one month later (OR: 1.07, 95 % CI: 1.01–1.13). We found no such association between second hand smoking campaigns either in the same month or at a two-month lag, and exposure to all other types of campaigns had no significant impact at any lag.

Adjustment for smoking prevalence did not change these effects, and specifically, the effect of second hand smoking campaigns at 1 month lag was unchanged (OR: 1.07, 95 % CI 1.01-1.14).

There was no evidence of modification of the effect of all campaigns, or specifically second hand smoking campaigns, in relation to either measure of socio-economic status either at 1 month lag (all campaigns NS-SEC: *p* = 0.7, IMD *p* = 0.2; second hand smoking campaigns NS-SEC: *p* = 0.4, IMD *p* = 0.11) or at other lags. There was also no significant interaction with the presence of a child in the home at 1 month lag (all campaigns: *p* = 0.6) or at other lags.

## Discussion

Televised tobacco control campaigns can change smoking behaviour [17, 18], but this is the first national study to investigate whether such campaigns can alter a smoker’s behaviour in the home. Our analyses show that, in those who continued to smoke, exposure to the varied mix of campaigns shown over recent years in England has not been associated with an increase in smoking restrictions in the home over and above the impact on smoking cessation. Campaigns with a specific second hand smoking theme have been limited in number, but our results provide an indication that such specifically targeted campaigns may have had some effect in reducing smoking in the home.

One limitation of our study was that household smoking behaviour was self-reported. It also used a different definition of smoke-free home ie no one smokes in the home on most days, from the more conservative definition of no one smoking at all in the home used in many studies. However, previous work in the Health Survey for England has demonstrated that in the subset of children with cotinine measurements of SHS exposure, the response to the question on smoking behaviour in the home is very strongly predictive of children’s cotinine levels [27] suggesting that this question does reflect relevant smoking behaviour. We used repeated cross-sectional surveys rather than longitudinal data. Moreover, GRPs are a population rather than an individual level of exposure. We were therefore unable to evaluate changes in smoking rules in individual households in relation to the household’s exposure; rather our findings are based on aggregate changes in the population over time in relation to estimated population levels of campaign exposure. This limited us to looking at short-term effects. Our results indicated that whether a home was smoke-free was strongly associated with season of the year, suggesting that the prevalence of smoke-free homes is influenced by short-term factors. Although we found an association with second hand smoking campaigns at 1 month lag, we found no association at 2 months lag which could indicate that any impact of the campaigns is short-lived. The small number of second hand smoke theme campaigns may explain why we did not find a stronger or longer lasting impact; 400 TVRs per month have been suggested to be needed to change smoking prevalence [17] and the exposure to second hand smoke campaigns was seldom anywhere close to this level. However, as we explored the impact of two different campaign themes at 3 lags and found one borderline significant result, it is also possible that the significant effect of campaigns with a second hand smoking theme at lag 1 may have arisen by chance, and our findings should be interpreted accordingly.

Nonetheless, the present study is the first of its kind to evaluate the impact of televised tobacco control campaigns on smoke-free homes using a large, country-wide sample. The patterns of associations of individual factors with having a smoke-free home in this study, including the composition of the household, age and gender of smokers within the household, the presence of children, occupation and socioeconomic status of the head of household are similar to those seen in other countries [5, 6]. The proportion of homes that were smoke-free increased over the period of this study, corresponding to similar trends in other countries [28, 29] and existing evidence from England [21]. The results of our multivariable models give some indication of an increase in smoke-free homes with increasing tobacco control score, and particularly with the introduction of smoke-free legislation (indicated in the tables by a rise in tobacco control score to 48). Our analysis has nevertheless allowed us to adjust for all of these individual factors, time trends and the growing strength of wider tobacco control policies in the UK over this time frame, and our results are therefore unlikely to be due to confounding. We have previously shown that televised tobacco control campaigns in England have made a small contribution to reductions in smoking prevalence [19], and it is therefore likely that they impact indirectly on the prevalence of smoke-free homes by encouraging smoking cessation; we therefore limited our sample to smokers in order to exclude any indirect effect occurring via reductions in smoking prevalence.

A review exploring the effects of population level interventions on smoke-free homes [14] found some direct evidence that comprehensive tobacco control programmes, including effective education, smoke-free places policies, and smoking cessation services, can increase the prevalence of smoke-free homes. However, it found only indirect support for other population-level interventions including mass media campaigns, based on the fact that those who believe SHS is harmful appear to be less likely to smoke in the home [6], and that mass media campaigns that have included SHS themes have been effective in increasing knowledge about the harms of SHS [15, 16]. A 1992 mass media campaign in Victoria, Australia, was found to have increased the proportion of non-smokers asking their visitors not to smoke, but seemed to have less effect on smokers [30]. In the USA, exposure to a media campaign on SHS resulted in increased intent to have smoke-free homes [15]. Previous studies from the UK showed that knowledge of SHS harms increased during 2003–2006 when more frequent SHS-related mass media campaigns were run compared to earlier years, and that smokers with better knowledge were more likely to have smoke-free homes [10]. Regional mass media campaigns promoting smoke-free homes were effective in increasing knowledge of the health impacts of SHS [31]. A small non-significant impact on the proportion of smoke-free homes was also seen but the study was underpowered. Our current study therefore provides the first tentative evidence that televised campaigns with a second hand smoking theme may be associated with an increase in smoke-free homes, at least in the month following the campaign. The lack of effect modification by socio-economic group provides some reassurance with respect to the concern that such population based interventions might potentially widen disparities in smoking through having less effect in more deprived groups; we found no evidence that this was the case though power for detecting interactions was inevitably low given the data available.

The theory around behaviour change and SHS has been reviewed by Borland [13]. This review advocates use of mass media firstly to increase knowledge and community-wide acceptance that second hand smoking is harmful, and once that is established, to promote control of SHS exposure in the home. Several recent studies from the UK suggest that there remains a lack of knowledge, and some resistance to the health messages, regarding the harms of smoking to others [10, 32] and that knowledge may be declining where mass media campaigns are not continued [31]; our findings therefore support the need for future mass media campaigns highlighting the dangers of SHS. We have recently shown in relation to mass media campaigns aimed at promoting smoking cessation that more positive messages providing information on how to quit are important alongside those showing the health consequences [33, 34]. If the same were true for second hand smoking campaigns, it may be helpful to include campaigns which show how smoke-free homes can be successfully achieved.

## Conclusion

There is considerable evidence of the harms of SHS exposure in children and other non-smokers living with a smoker, and evidence that living in a smoke-free home is also beneficial to the smoker who is more likely to quit smoking. However, many homes with a smoker are not smoke-free. Our findings suggest that televised media campaigns promoting smoking cessation may not be effective in reducing smoking in the home, but we found tentative evidence that campaigns specifically targeting second hand smoke may do so. Further use of this type of campaign, with appropriate evaluation to confirm its effectiveness, would be appropriate.
